# 

**Published:** 1889-09-14

**Authors:** 


### NOTICE TO CORRESPONDENTS.

All MS., Letters, Books for Review, and other matters intended for the Editor, should be addressed The Editor, The Lodge, Porchester Square, London, W.

### Notice to Advertisers and Subscribers.

All Advertisements, Orders for Copies of The Hospital, and Business Communications should be addressed to The Publisher, *not to the Editor*, The Hospital, 139-140, Salisbury-court, Fleet-street, E.C.

Vol. V., now ready, 4s., post free. Cases for binding, may be had of the Publisher, at 1s. 6d. each.

### To Residents, Secretaries, and Matrons.

The Editor will be glad to have the Regulations and each Report as issued by Asylums, Hospitals, Convalescent and Nursing Institutions, as well as those of other Charities, and an early copy in duplicate of all Appeals and Festival Notices, etc., addressed to *The Editor, The Lodge, Porchester-square, London, W.* Any superintendent, secretary, matron, or other chief official who regularly sends such information may be r egistered, on application to the Publisher, to receive free of cost a cover or binding The Hospital in half-yearly volumes.

### AMUSEMENTS AND RELAXATION.

### Second Quarterly Word Competition Commenced July 6th, ends September 28th.

Three Prizes of 15s., 10s., 5s., will be given for the largest number of words derived from the words set for dissection.

Proper names, abbreviations, foreign words, words of less than four letters, and repetitions are barred; plurals, and past and present participles of verbs, are allowed. Nuttall's *Standard* dictionary only to be used.

N.B.—Word dissections must be sent in WEEKLY, arranged alphabetically, with correct total affixed.

The word for dissection for this, the *Eleventh* week of the quarter, being "DONCASTER."

### Results of Ninth Week.

| Names. | Sept. 5th. | Totals. | Names. | Sept. 5th. | Totals. |
|---|---|---|---|---|---|
| Nurse Duty | 15 | 496 | Leirion | — | 195 |
| N'importe Qui | 14 | 454 | Matron | — | 174 |
| Jumps | — | 30 | F. C. J. | — | 305 |
| Tinie | — | 186 | Percy Verance | — | 18 |
| Patience | 15 | 502 | Gleniffer | — | 175 |
| Amicus | — | 430 | Gypsye | 15 | 437 |
| Broxbourne | — | 398 | Ladyr | — | 127 |
| Speedwell | 15 | 454 | A. B. | — | 170 |
| Sister | 14 | 477 | Essie | 13 | 393 |
| Lightowlers | — | 356 | W. G. R. | 13 | 388 |
| Lauriston | — | 345 | Cowboy Bill | — | 179 |
| Rattler | — | 28 | M. L. A. | — | 117 |
| Paignton | — | 334 | Little Tuppy | — | 110 |
| Fassifern | — | 141 | Embryo | — | 56 |
| W. R. E. | 15 | 483 | K. Jarman | — | 43 |
| Esperance | 15 | 495 | Reveal | — | 196 |
| M. W. | 15 | 500 | Jenny Wren | 15 | 387 |
| Isabel | 15 | 469 | Condorcet | 14 | 420 |
| Buxton | — | 167 | Electrician | 15 | 260 |
| Nux Vom | — | 263 | Pearl | — | 132 |
| Qu'appelle | 14 | 433 | R. W. | — | 43 |
| Spes | — | 24 | Nurse Amie | — | 89 |
| Secnarf | — | 229 | Sunshine | 14 | 176 |
| Rover | — | 226 | Juvenis | — | 23 |

### Notice to Correspondents.

N.B.—All letters referring to this page which do not arrive at 139 and 140, Salisbury-court, London, E.C., by the *first post on Thursdays*, and are not addressed PRIZE EDITOR, will in future be disqualified and disregarded

Competitors can enter for *all* quarterly competitions, but no competitor may take more than *two quarterly prizes* during the year.

